# Research on Low-Damage CO_2_ Foam Flooding System: Review and Outlook

**DOI:** 10.3390/molecules31040642

**Published:** 2026-02-12

**Authors:** Jierui Liu, Zhen Cui, Shisheng Liang, Xinyuan Zou, Wenli Luo, Wenjuan Wang, Bo Dong, Xiaohu Xue

**Affiliations:** 1 PetroChina Research Institute of Petroleum Exploration & Development, Beijing 100083, China; zouxy2016@petrochina.com.cn; 2 State Key Laboratory of Enhanced Oil and Gas Recovery, Beijing 100083, China; 3 China Petroleum Technology and Development Corporation, Beijing 100028, China; cuizhen01@cnpc.com.cn (Z.C.); xuexh@cnpc.com.cn (X.X.); 4 Sinopec Research Institute of Petroleum Engineering (Tianjin) Technology Development Co., Ltd., Tianjin 300280, China; liang405.sripe@sinopec.com; 5 No. 1 Gas Production Plant, SINOPEC North China Oil and Gas Company, Zhengzhou 450006, China; wangwenjuan199@163.com; 6 University of Chinese Academy of Sciences, Beijing 100049, China; dongbo22@mails.ucas.ac.cn; 7 Institute of Porous Flow and Fluid Mechanics, Chinese Academy of Sciences, Langfang 065007, China

**Keywords:** strongly water-sensitive, tight oil reservoirs, CO_2_ foam flooding, clay stabilizers, organic emulsion foam, CO_2_-soluble surfactants

## Abstract

Tight oil reservoirs are widely recognized as a critical successor in global unconventional energy development and are generally characterized by distinct geological features, including fine pore throats, pronounced heterogeneity, and a high concentration of clay minerals (e.g., montmorillonite and mixed-layer illite/smectite). Severe hydration, swelling, and fines migration are readily induced during water injection or conventional water-based fluid operations, thereby resulting in irreversible impairment of reservoir permeability. Despite the excellent injectivity and capacity for viscosity reduction associated with CO_2_ flooding, sweep efficiency is severely compromised by viscous fingering and gas channeling, which are induced by the inherent low viscosity of the gas. While CO_2_ foam technology is widely acknowledged as a pivotal solution for addressing mobility control challenges, its implementation is hindered by a primary technical bottleneck: the incompatibility between traditional water-based foam systems and strongly water-sensitive reservoirs. A dual challenge comprising water injectivity constraints and gas channeling is presented by strongly water-sensitive tight oil reservoirs. To address these impediments, three emerging low-damage CO_2_ foam systems are critically evaluated in this review. First, the synergistic mechanisms of novel quaternary ammonium salts and polymers in inhibiting clay hydration and enhancing foam stability within modified water-based systems are elucidated. Next, the physical isolation strategy of substituting the water phase with a non-aqueous phase (oil/organic solvent) in organic emulsion systems is analyzed, highlighting advantages in wettability alteration and the mitigation of water blocking. Finally, the prospect of waterless operations using CO_2_-soluble foam systems—wherein supercritical CO_2_ is utilized as a surfactant carrier to generate foam or viscosify fluids via in situ formation water—is discussed. It is revealed by comparative analysis that: (1) Modified water-based systems are identified as the most economically viable option for reservoirs with moderate water sensitivity, wherein cationic stabilizers are utilized to inhibit hydration; (2) Superior wettability alteration and the elimination of aqueous phase damage are provided by organic emulsion systems, rendering them ideal for ultra-sensitive, high-value reservoirs, despite higher solvent costs; (3) CO_2_-soluble systems are recognized as the future direction for “waterless” flooding, specifically tailored for ultra-tight formations (<0.1 mD) where injectivity is critical. Current challenges, such as surfactant solubility, high-temperature stability, and cost control, are identified through a comparative analysis of these three systems with respect to structure-activity relationships, rheological properties, damage control capabilities, and economic feasibility. What is more, an outlook is provided on the molecular design of future environmentally sustainable, cost-effective CO_2_-philic materials and smart injection strategies. Consequently, theoretical foundations and technical support are established for the efficient exploitation of strongly water-sensitive tight oil reservoirs. By bridging the gap between reservoir damage control and mobility enhancement, this study identifies viable strategies for enhanced oil recovery. Crucially, it supports carbon neutrality and sustainable energy targets via CCUS integration.

## 1. Introduction

Against the backdrop of rapid global economic and social development, a continuous escalation in energy demand is observed. As petroleum remains a dominant energy source among fossil fuels, the efficient extraction of crude oil is rendered critical for satisfying energy requirements. Consequently, within the petroleum industry, the development of unconventional reservoirs has emerged as a strategic focal point. It is widely acknowledged that unconventional oil and gas resources possess substantial development potential and can play a vital role in effectively mitigating energy shortages [1,2,3,4,5]. Specifically, the proven reserves of tight oil globally stand at 639.3 billion tons [6], indicating vast development prospects for the next half-century. However, tight oil reservoirs are typically characterized by pronounced water sensitivity and low to ultra-low permeability (<1 mD). Consequently, severe challenges for water and gas flooding technologies are presented by the high argillaceous content, abundant clay minerals, and significant heterogeneity inherent in the rock matrix [7,8,9,10,11]. When water or steam flooding is employed, the swelling of clay minerals is readily induced by the introduction of extraneous water. This phenomenon leads to pore throat blockage, permeability impairment, and formation instability, thereby causing significant formation damage and exacerbating operational challenges. In contrast, gas flooding is frequently compromised by severe gas channeling, resulting in diminished sweep efficiency; consequently, recovery factors are often observed to remain below 20% [12,13,14].

CO_2_ flooding is widely recognized as a prevalent tertiary oil recovery method. Its capability to significantly enhance oil recovery in low-permeability and tight reservoirs has been extensively demonstrated. Recovery is enhanced by CO_2_ through mechanisms such as the extraction of light hydrocarbons to induce crude oil swelling, the significant reduction in oil viscosity, the improvement of the oil–water mobility ratio, the reduction in interfacial tension, and the achievement of miscibility with reservoir oil [15,16]. Xian et al. [17] systematically investigated the factors influencing the oil recovery efficiency of CO_2_ flooding. Their studies indicate that, for medium-viscosity crude oil, elevated pressure enhances the solubility of CO_2_ in the oil, thereby improving its fluidity. Increasing the CO_2_ injection rate can further boost oil recovery; however, it may lead to premature gas breakthrough. Moreover, significant reservoir heterogeneity markedly reduces the effectiveness of CO_2_ flooding and accelerates gas channeling. Chen et al. [18] demonstrate that miscibility is the most critical factor influencing the effectiveness of CO_2_ flooding. Under miscible conditions, CO_2_ and crude oil merge into a single-phase flow, eliminating interfacial tension and thereby maximizing displacement efficiency, while the impact of gravity segregation becomes less pronounced. In contrast, under immiscible conditions, displacement efficiency is considerably lower, and gravitational effects play a more significant role. Zhang [19] et al. employed in situ CT scanning and digital rock technology, integrated with machine learning approaches, to systematically investigate the effects of flow patterns, residual oil distribution, and pore structure in porous media on oil displacement efficiency during water flooding and CO_2_ flooding. The results indicate that increasing the throat radius reduces capillary resistance at the gas-oil interface and enhances CO_2_ displacement efficiency; however, it may also lead to premature CO_2_ breakthrough. Wang [20] et al. verified that an increase in reservoir thickness intensifies CO_2_ gravity override, resulting in gas segregation at the top, poor oil mobilization at the bottom, and a consequent reduction in oil displacement efficiency. Furthermore, it was observed that stronger heterogeneity and larger permeability contrasts lead to more severe CO_2_ channeling. This results in distinct preferential flow paths, lower sweep efficiency, and a significant decline in the recovery rate. Concurrently, preferential flow paths are established by large channels and fractures, allowing CO_2_ to bypass oil zones and break through prematurely. Consequently, sweep efficiency in the matrix is substantially diminished, ultimately lowering the final recovery factor. The crude oil production characteristics of CO_2_ flooding in tight reservoirs, along with the factors influencing displacement efficiency, were investigated by Wang et al. [21] While the CO_2_ miscible flooding effect was found to be significant in medium-sized pore throats, it was determined that auxiliary measures must be employed in large pore throats to suppress gas breakthrough. In general, it is indicated that the exclusive application of CO_2_ flooding may result in challenges such as gas channeling and low sweep efficiency. While conventional plugging and profile control methods employed in conjunction with CO_2_ flooding—such as gels and water-based foam systems—are effective in conventional reservoirs, they are deemed unsuitable for strongly water-sensitive tight oil reservoirs. Furthermore, given that current research is predominantly focused on foams for conventional reservoirs, an urgent need exists for the improvement and optimization of CO_2_ foam flooding formulations specifically designed to inhibit water sensitivity. As an efficient tertiary oil recovery technology, the dual advantages of CO_2_ flooding and foam flooding are synergistically combined within the CO_2_ foam flooding process [22]. During this process, gas mobility is significantly reduced by the foam through an increase in the apparent viscosity of the gas; consequently, viscous fingering and gravity override are effectively inhibited. Furthermore, oil–water interfacial tension (IFT) and capillary forces are lowered by the surfactants present in the foam, which facilitates the mobilization of trapped residual oil. Additionally, rock wettability is altered from oil-wet to water-wet, thereby enhancing displacement efficiency [23,24]. The significant application potential of CO_2_ foam flooding in both high and low permeability reservoirs has been confirmed through numerous laboratory core flooding experiments. Furthermore, its feasibility in ultra-low permeability tight oil reservoirs has been demonstrated, wherein effective mobility control is provided by the CO_2_ foam [25]. However, the in situ propagation and stability of CO_2_ foam are significantly governed by reservoir and geological parameters. Critical challenges—including temperature, formation pressure, salinity, water sensitivity, and reservoir heterogeneity—must be effectively addressed; consequently, the screening and formulation of suitable foam stabilizers are necessitated to ensure long-term stability [26,27,28,29]. It is observed that enhanced recovery rates exhibit significant variation depending on the injection mode employed; specifically, the efficiency limitations associated with foam injection can be effectively mitigated by its combination with surfactant-polymer flooding, most notably in reservoirs where pressure exceeds the minimum miscibility pressure (MMP) [30]. Moreover, from an environmental standpoint, not only is oil recovery enhanced by CO_2_ foam flooding technology, but effective CO_2_ sequestration is also facilitated, thereby providing theoretical and technical support for CCUS-EOR and yielding dual economic and environmental benefits [31,32,33,34].

In summary, given the conditions characteristic of strongly water-sensitive tight oil reservoirs, the optimization and innovation of traditional CO_2_ foam flooding technology are deemed critical for the effective exploitation of its unique advantages in enhanced oil recovery. Through the advancement of research and displacement technologies, it is anticipated that the application scope of CO_2_ foam flooding will be expanded, thereby providing substantial support to the field of EOR. Although several reviews have discussed foam stability or CO_2_ EOR mechanisms separately, there is a notable gap in the literature regarding the compatibility paradox specific to strongly water-sensitive tight reservoirs. Existing reviews often overlook the critical conflict where the water phase required for conventional foam generation is the very agent triggering clay damage. Furthermore, a systematic framework for selecting between water-based, organic-based, and CO_2_-soluble systems based on reservoir damage severity is currently absent. This review aims to bridge these gaps. In this review, a comprehensive overview is provided regarding the development challenges associated with strongly water-sensitive tight oil reservoirs, the mechanisms of water sensitivity, the research progress of CO_2_ foam flooding in EOR, and relevant field applications. Concurrently, based on the current status of oilfield development in strongly water-sensitive formations, three technical routes designed to mitigate water sensitivity are systematically outlined: (1) novel systems incorporating clay stabilizers into conventional water-based CO_2_ foam; (2) organic CO_2_ emulsion foam systems; and (3) CO_2_-soluble foam systems. Finally, current limitations and future development directions are discussed with the aim of promoting the application of CO_2_ foam flooding in strongly water-sensitive reservoirs. This analysis serves to facilitate further improvements in crude oil recovery while offering feasible solutions for geological CO_2_ sequestration.

## 2. Mechanism and Evaluation of Water Sensitivity Damage in Strongly Water-Sensitive Tight Reservoirs

Strongly water-sensitive tight oil reservoirs are recognized as a pivotal category within unconventional resource development. While unique geological and fluid characteristics are exhibited by these reservoirs, numerous technical bottlenecks are encountered during their development. The primary difficulty is identified as the control of reservoir damage during extraction. Compositionally, these reservoirs are typically characterized by high concentrations of clay minerals. Consequently, the mitigation or inhibition of clay mineral swelling is regarded as the pivotal aspect of developing such reservoirs. The elucidation of the structure and properties of reservoir clay minerals, alongside the adoption of targeted technical countermeasures, is deemed essential for efficient development and utilization [35,36].

### 2.1. Main Types and Characteristics of Clay Minerals

Clay minerals are layered aluminosilicates primarily composed of silica tetrahedra and alumina octahedra sheets. In tight oil reservoirs, the most common clay minerals include kaolinite, montmorillonite [37,38,39], illite [37,40,41], chlorite [40,42], and mixed-layer clays (e.g., I/S) [37,40,43,44]. Their swelling potential is dictated by their crystal lattice structure and Cation Exchange Capacity (CEC) (Table 1). Unlike non-swelling kaolinite and illite, montmorillonite and mixed-layer I/S possess weak interlayer bonding forces (van der Waals), allowing water molecules to enter the interlayer space, leading to significant crystalline swelling [45,46].

### 2.2. Properties of Clay Minerals

#### 2.2.1. Electrical Charge

The surface charge of clay minerals is defined by the sign and magnitude of the electrostatic charge exhibited upon contact with an aqueous phase. This charge is attributed to two primary sources, the first of which involves the dissociation of exchangeable cations. A specific abundance of exchangeable cations is inherent to clay mineral surfaces. Upon hydration, these cations dissociate from the surface. Subsequently, they are distributed via diffusion to form a diffuse double layer. This process ultimately renders the clay surface negatively charged. Consequently, a greater density of exchangeable cations correlates with an elevated surface charge. This results in a more pronounced negative potential following dissociation. The magnitude of this surface charge is quantitatively expressed by the Cation Exchange Capacity (CEC) [47], defined as the total amount of cations (expressed in moles of monovalent cations) that can be exchanged from 1 kg of clay mineral at pH 7, with units of mmol·kg^−1^. Different clay types have different CECs (Table 1).

**Figure 1:**
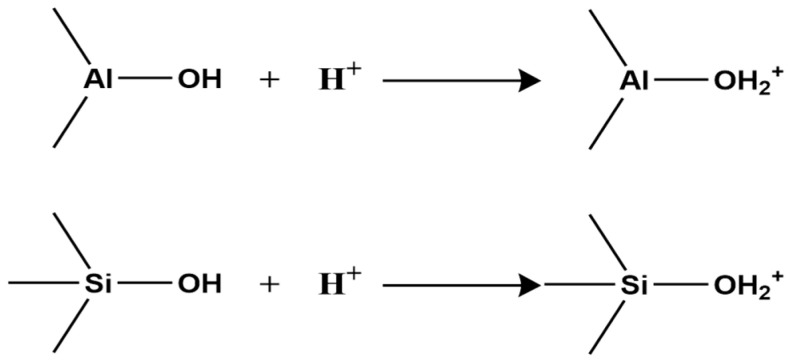
Surface Hydroxyl Generation Process.

Second, clay minerals contain two types of surface hydroxyl groups: those on the crystal layer surface and those at broken edges reacting with H^+^ and OH^−^ in water (Figure 1).

**Figure 2:**
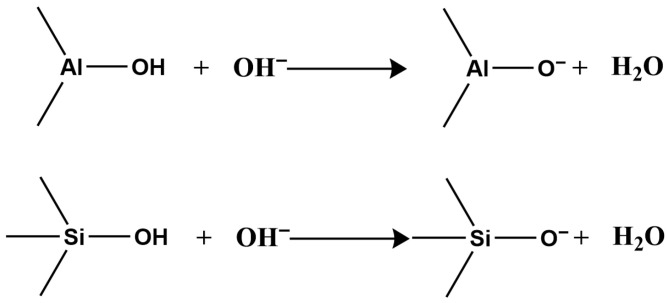
Reaction under acidic conditions.

**Figure 3:**
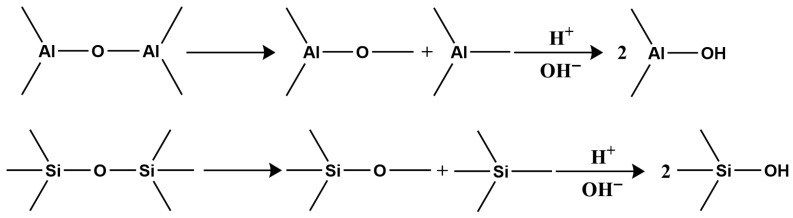
Reaction under alkaline conditions.

In acidic or alkaline conditions, these surface hydroxyls react with H^+^ or OH^−^, altering the surface charge sign. Under acidic conditions, hydroxyls react with H^+^, making the surface positively charged (Figure 2);

Under alkaline conditions, they react with OH^−^, making the surface negatively charged (Figure 3).

The algebraic sum of charges from these two sources determines the final charge of the clay mineral. Generally, clay surfaces are negatively charged.

#### 2.2.2. Adsorption Mechanisms

Adsorption on clay surfaces is driven by electrostatic and hydrophobic interactions. Non-ionic surfactants adsorb primarily via hydrogen bonding (Physical Adsorption) [40].

For cationic surfactants (e.g., quaternary ammonium salts), the primary mechanism is Ion Exchange and Electrostatic Attraction with the negatively charged clay surface. While traditionally considered strong adsorption, recent studies indicate that this process is reversible to a certain extent, depending on pH and ionic strength, rather than forming permanent chemical bonds [48,49]. This reversibility is a critical factor in the long-term effectiveness of clay stabilizers.

#### 2.2.3. Swelling

Significant variation is observed in the swelling properties of clay minerals. Based on crystal structure, clay minerals are categorized into swelling and non-swelling types. Montmorillonite is classified as a swelling clay; its swelling behavior is attributed to the abundance of exchangeable cations. Upon contact with the aqueous phase, the interlayer space is infiltrated by water molecules, resulting in the dissociation of cations and the establishment of a diffuse double layer, thereby generating negative polarity. The interlayer spacing is expanded by the repulsion between negatively charged layers, a phenomenon manifested as swelling.

In contrast, kaolinite, illite, and chlorite are designated as non-swelling minerals. Significant lattice substitution is absent in kaolinite, while the presence of interlayer hydrogen bonds is observed; in illite, lattice substitution is primarily located in the tetrahedral sheet, wherein layers are tightly bound by potassium ions (K^+^); finally, chlorite is characterized by interlayer hydrogen bonds and brucite-like sheets that serve to compensate for charge imbalances [50].

### 2.3. Complexity of CO_2_–Water–Rock Interactions

Upon CO_2_ injection, carbonic acid is formed through the interaction with formation water, resulting in a significant reduction in pH. Subsequent water-rock interactions are induced by this acidification. The initial reaction of acidic fluids occurs with the most reactive minerals, specifically carbonate cements (e.g., calcite, dolomite) [51]. In tight reservoirs, the cementation or encapsulation of clay minerals (e.g., kaolinite) by carbonate phases is frequently observed [52]. However, this structural integrity is compromised by the rapid dissolution of carbonate. This dissolution triggers the release of fine particles, which subsequently impede pore throats. Specifically, authigenic clay particles (particularly kaolinite) and quartz microcrystals were previously immobilized on pore walls. However, they are liberated by this dissolution process [53]. Driven by the aqueous phase of the foam, these fines migrate and eventually obstruct narrower downstream pore throats; consequently, a drastic and irreversible decline in permeability is precipitated [54,55]. Dissolution acts as a catalyst for “water sensitivity damage.”

CO_2_ foam injection generates the Jamin effect [56]. In porous media, foam is characterized as a discontinuous phase of discrete gas bubbles exhibiting high apparent viscosity. Its mobility control mechanism is fundamentally predicated on the macroscopic manifestation of the Jamin effect [57]. As foam flows through porous media, numerous liquid films (lamellae) must overcome capillary resistance at pore throats to deform and pass. According to Hirasaki and Lawson, the pressure gradient (▽Pcap) required is proportional to interfacial tension (γ) and inversely proportional to pore radius (rt) [58]. In conventional reservoirs, the Jamin effect is positive, providing necessary resistance for profile control. However, in tight reservoirs characterized by micro- or nano-scale pore radii, the Jamin effect is drastically amplified (▽Pcap ∝ 1/rt). Consequently, an extremely high pressure gradient is necessitated for effective foam propagation [59]. This phenomenon is deemed detrimental to strongly water-sensitive reservoirs, wherein the structural integrity of the rock matrix is already compromised by clay hydration. High hydrodynamic forces are applied to a mechanically weakened framework. This precipitates mechanical shearing and stripping, rather than achieving effective profile control. Consequently, hydrated clay particles are detached from pore walls, culminating in pore collapse and fragmentation [60,61]. Therefore, system design must prioritize avoiding the negative impacts of the Jamin effect [62].

### 2.4. Characterization and Evaluation of Strong Water Sensitivity Damage

Characterizing damage is a multi-scale challenge involving static identification and dynamic quantification of fluid-rock interactions [63].

#### 2.4.1. Static Evaluation

Static evaluation rapidly identifies water-sensitive minerals and their occurrence to assess risk [64]. X-ray Diffraction (XRD) analyzes mineral composition: whole-rock XRD provides macro ratios of skeletal minerals (quartz, feldspar, carbonate); clay fraction (<2 μm) XRD precisely identifies clay types and contents, especially swelling montmorillonite and I/S mixed layers [65]. Given that sample crushing is required for XRD analysis, the supplementation of Scanning Electron Microscopy (SEM) and Energy Dispersive Spectroscopy (EDS) is necessitated to observe micro-morphology and spatial occurrence (e.g., pore-lining, pore-filling, and pore-bridging forms). The Free Swell Test is employed as a direct, semi-quantitative method for measuring the swelling volume of crushed samples in various fluids. While effective for the screening of stabilizers, the absence of confining pressure in this method is noted; consequently, swelling rates that significantly exceed those observed under actual reservoir conditions are produced [66].

#### 2.4.2. Dynamic Evaluation

The quantitative characterization of formation damage and the evaluation of stabilizer effectiveness are accomplished through core flooding experiments [64]. The monitoring of permeability (K) variations constitutes the central objective of this experimental phase. Initially, a baseline permeability (Kb) is determined under simulated reservoir conditions through the injection of an “inert” fluid (e.g., high-salinity KCl brine or oil). Subsequently, the injection stream is transitioned to a “damaging” fluid. Finally, the permeability damage rate (D%) is calculated as follows [67]:

D% = (Kb − Kd)/Kb × 100%

The Critical Salt Concentration (CSC) is determined through the gradual reduction in salinity.

Additionally, the occurrence of “Fines Migration” is confirmed through the analysis of the effluent using laser particle size analyzers or turbidity meters; specifically, the observation of peaks in particle concentration following fluid switching serves as a definitive indicator.

#### 2.4.3. Visualization Technology

Dynamic experimental evaluations are complemented by visualization techniques, thereby facilitating the elucidation of the spatial distribution and microscopic mechanisms associated with water-induced damage [68].

X-ray Computed Tomography (CT) is utilized to facilitate the 3D/4D imaging of internal pore architectures. Through this technique, the monitoring of foam propagation fronts, fluid saturation distributions, and profile control efficacy is effectively achieved [69]. In the context of water sensitivity studies, the accumulation of particles at pore throats or the formation of wormholes is effectively visualized.

Nuclear Magnetic Resonance (NMR): Pore structure characteristics are reflected by the T_2_ relaxation times of hydrogen protons. This technique is proven effective for the characterization of both swelling (where reduced pore radii correspond to shortened T_2_ values) and fines migration (indicated by signal loss in large pores due to blockage) [70].

## 3. Modified Water-Based Anti-Swelling CO_2_ Foam Flooding System

### 3.1. Principle of System Construction

While water is the indispensable carrier for conventional foam [28,71,72,73,74], its interaction with clay minerals poses significant risks. Unlike conventional systems, the modified water-based system focuses on chemical inhibition rather than physical isolation, utilizing stabilizers to maintain lattice spacing as detailed in Section 2.

The adoption of a water-based system incorporating clay stabilization functions is regarded as the most direct solution. The fundamental principle involves not the elimination of the aqueous phase, but rather its effective “passivation.” Furthermore, significant cost-effectiveness and technological maturity are offered by water-based foam systems. To counteract the cation exchange capacity (CEC) issues mentioned in Section 2.2.1. This strategy is implemented through the incorporation of efficient clay stabilizers into the aqueous phase. The aqueous phase is utilized as a carrier vehicle by which chemical agents are transported deep into the reservoir matrix; therein, preferential adsorption onto active clay sites is facilitated. Consequently, upon contact between foam lamellae and pore walls, a protective state is already established within the formation, thereby ensuring “low damage” displacement.

### 3.2. Clay Stabilizers

Stabilizers are common in drilling and fracturing [75,76,77]. However, the application of these agents within long-term CO_2_ foam EOR is recognized as a formidable challenge, necessitating properties of irreversible adsorption and effective transportability.

#### 3.2.1. Inorganic Salts

Inorganic salts like KCl, NH_4_Cl, and CaCl_2_ are first-generation stabilizers [73]. The silicate “hexagonal cavities” are occupied by cations possessing specific ionic radii (e.g., K^+^, 0.133 nm), whereby the crystal layers are effectively immobilized via electrostatic attraction [78]. High ionic strength compresses the electrical double layer. However, they have fundamental defects for long-term EOR: high dosage requirements (2–7% KCl) increase costs, and the adsorption is reversible. K^+^ ions are gradually washed away (Leaching) during long-term flooding, causing failure [79,80].

#### 3.2.2. Organic Small Molecule Amines/Quaternary

Ammonium Salts represented by short-chain (TMAC), long-chain (CTAB, TMA), and novel Choline Chloride [81], these are second-generation agents. Cationic heads anchor to negative clay surfaces via electrostatics, while hydrocarbon chains extend into the water [75]. The operative mechanisms are categorized as follows: (1) Crystal layer intercalation, which serves to neutralize surface charges and stabilize the lattice structure; and (2) Hydrophobic surface modification, wherein the ingress of the aqueous phase is effectively repelled through the formation of a protective hydrophobic film [82]. Superior efficiency (typically at dosages of 0.1–0.5%) and enhanced adsorption capabilities are exhibited by these agents. Furthermore, while biological toxicity is typically associated with traditional long-chain quaternary ammonium compounds (“quats”), a green and biodegradable alternative is provided by Choline Chloride [83]. A functional mechanism analogous to that of KCl is exhibited by short-chain salts such as Tetramethylammonium Chloride (TMAC); however, these agents are distinguished by significantly stronger and more irreversible adsorption characteristics. Furthermore, crucial importance is assigned to compatibility with foaming agents; specifically, precipitation is induced by the interaction between cationic stabilizers and anionic surfactants (prevalent foaming agents), whereby both foaming and stabilizing functionalities are effectively neutralized [84].

#### 3.2.3. High Molecular Weight Polymers

The class of cationic polymers is exemplified by agents such as cationic polyacrylamide (CPAM), polyamines, polydiallyldimethylammonium chloride (Poly-DADMAC), and cationic guar gum (CPG) [85,86]. Classified as third-generation high-efficiency anti-swelling agents, distinct advantages within the context of EOR engineering are offered by these polymers. A high density of cationic moieties (e.g., quaternary ammonium and amine groups) is exhibited along the polymer backbone, facilitating robust, multi-site adsorption onto negatively charged clay surfaces. Specifically, simultaneous adsorption onto multiple clay particles or platelets (bridging) is achieved by a single polymer chain, resulting in the consolidation of loose particulates; alternatively, complete encapsulation (coating) of individual particles is effected, ensuring their isolation from the aqueous phase.

High molecular weight polymers are considered theoretically optimal candidates for EOR anti-swelling applications; this distinction is attributed to their nearly irreversible adsorption characteristics, high resistance to leaching, and the requirement for only minimal dosages (typically 500–2000 ppm) [87]. However, the application of these polymers in tight reservoirs is hindered by a fundamental physical contradiction. First, to ensure efficient bridging and coating mechanisms, a significantly high molecular weight (typically M_w_ > 10^5^ Da) is necessitated. The application of high-molecular-weight (M_w_) polymers in tight reservoirs is associated with significant risk. Given that pore throat radii frequently exhibit micrometer- to nanometer-scale dimensions, bridging and retention are readily induced by large polymer coils. Consequently, pore plugging and a precipitous decline in permeability are precipitated [88]. Through this mechanism, the polymer is inadvertently transformed from an intended “anti-swelling agent” into a detrimental “plugging agent.” Consequently, while the mitigation of chemical damage is achieved, the induction of more severe physical damage is simultaneously precipitated. It was explicitly confirmed by Liao et al. [88] that in tight cores, the permeability damage rate associated with high-molecular-weight polymer stabilizers exhibits a positive correlation with increasing polymer molecular weight (M_w_). Consequently, the selection of clay stabilizers possessing relatively lower molecular weights is necessitated, provided that the requirements for clay mineral anti-swelling remain satisfied. Secondly, excellent compatibility between the selected polymer and the foaming agent is required; specifically, the maintenance of optimal comprehensive foam performance (i.e., foaming volume and drainage half-life) upon the combination of these components is essential. Finally, high-molecular-weight polymers exhibit robust adsorption. However, the susceptibility of the polymer backbone (e.g., polyacrylamide) to hydrolysis or thermal degradation is acknowledged under acidic conditions and temperatures exceeding 90 °C. Consequently, a temporal decay in anti-swelling efficacy is often precipitated.

### 3.3. Selection of Foaming Agents

Vital importance is attributed to the synergy between foaming agents and stabilizers. However, it is noted that while cationic character is exhibited by the most efficient stabilizers, prevalent foaming agents (e.g., AOS, SDS) are anionic in nature [28,71,72,73,74]; consequently, precipitation is frequently induced by the interaction of these components [84,89]. Compatibility and performance metrics (e.g., IFT reduction and stability) must be ensured via rigorous screening protocols, particularly under reservoir conditions characterized by high salinity, elevated temperatures, and the presence of CO_2_. It was reported by Bello [90] that crude oil recovery was increased to 96% through the utilization of CO_2_ foam generated by a non-ionic binary surfactant system, whereas a recovery rate of 85% was obtained via CO_2_ flooding alone. Furthermore, enhanced foam stability and reduced rock adsorption (characterized by an 89.26% reduction in adsorption mass) were exhibited by this system within high-temperature and high-salinity carbonate rock environments. A cationic surfactant formulation suitable for enhanced CO_2_ foam flooding in high-temperature, high-salinity, and high-hardness carbonate reservoirs was developed by Gland [91]. Through this development, adsorption onto carbonate rocks was significantly reduced (with static adsorption tests indicating a reduction of greater than ≈50%), thereby mitigating the adsorption challenges attributed to the positively charged surface of carbonate formations. Moreover, robust solubility and foam stability were maintained by the optimized cationic formulation (F-OPT), even under conditions of high temperature 80 °C) and high salinity (TDS reaching 163 g/L). A class of ethoxylated cationic surfactants (e.g., ethoxylated coco amine) was successfully developed by Chen et al. [92]; these agents demonstrated the capacity to stabilize CO_2_/water foam under reservoir conditions characterized by high temperature (120 °C) and high salinity (up to 182 g/L NaCl). Consequently, the susceptibility of traditional surfactants to failure within such extreme environments was effectively mitigated. Due to the cationic nature of these surfactants, reduced adsorption on carbonate rock surfaces (e.g., calcite) is facilitated via electrostatic repulsion; conversely, while higher adsorption is observed on silica-containing impurities (such as clay within dolomite), this phenomenon is further inhibited by high salinity conditions (22% TDS). Novel Gemini betaine surfactants were successfully synthesized by Xiao et al. [93]. Through the synergistic combination of the structural advantages inherent to both Gemini surfactants and betaine, a series of sulfonated Gemini betaine surfactants featuring varying hydrophobic chain lengths (specifically BGS-12, BGS-14, and BGS-16) were designed. Consequently, long-term foam stability within high-salinity and high-temperature environments was achieved, thereby demonstrating both synthesis controllability and significant application potential.

Drawing upon established research, the selection of foaming agents from the categories of non-ionic, cationic, and amphoteric surfactants is recommended to ensure optimal compatibility with clay stabilizers. Within the scope of this research, the utilization of these existing surfactant classes is proposed; alternatively, the design, synthesis, or molecular modification of novel surfactants may be pursued to attain superior foaming performance and anti-adsorption capabilities.

### 3.4. Performance Evaluation Methodology

**Figure 4:**
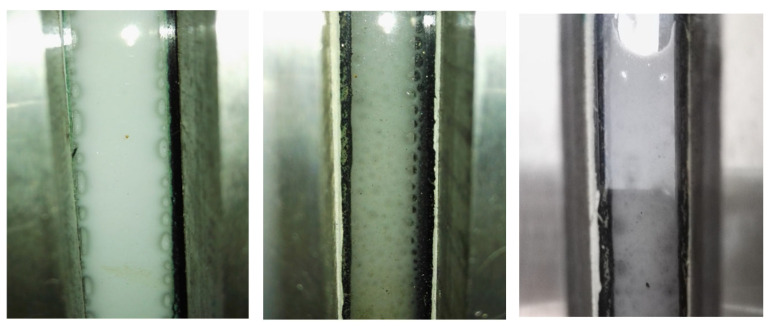
Morphology of modified water-based anti-swelling CO_2_ foam observed using a foam analyzer.

The evaluation of foam stability is typically executed via a multi-scale approach, encompassing a transition from static screening to dynamic rheological testing. For initial screening, the Waring Blender or Ross-Miles method is frequently utilized to quantify foam volume (V_0_) and half-life (t_1/2_) under ambient conditions [94]. The macroscopic morphology of the modified water-based anti-swelling CO_2_ foam was evaluated using a foam analyzer (The equipment manufacturer is PetroChina Research Institute of Petroleum Exploration & Development, Beijing, China). As shown in Figure 4, the generated foam exhibits a dense and uniform texture, indicating excellent stability under high-temperature conditions. While a rapid assessment of foaming capacity is provided by these tests, their inadequacy in simulating the high-temperature and high-pressure (HTHP) conditions characteristic of deep reservoirs is acknowledged. Consequently, the employment of HTHP rheometers is deemed essential for the evaluation of foam behavior under realistic shear conditions. Through this instrumentation, the apparent viscosity of the foam is measured under varying shear rates to facilitate the determination of its shear-thinning behavior.

The fluid diversion capacity of foam is quantified via the Mobility Reduction Factor (MRF) or Resistance Factor (RF). Specifically, the effective reduction in gas mobility via the Jamin effect is indicated by a high MRF value, whereby an improvement in sweep efficiency is subsequently facilitated [95,96]. In contrast to conventional evaluation protocols, the monitoring of the Permeability Damage Rate (D_k_) is necessitated during the assessment of anti-swelling systems [67]. According to standard protocol, baseline permeability (K_0_) is first determined utilizing a non-reactive fluid (e.g., kerosene or high-salinity brine), followed by the injection of the foam liquid phase. Subsequently, the effective inhibition of clay hydration alongside the assurance of injectivity is confirmed by a low D_k_ value (<10%) combined with a stable pressure build-up, in contrast to the sharp pressure spikes characteristic of pore plugging.

CO_2_ foam flooding experiments were performed utilizing oil-saturated cores derived from strongly water-sensitive tight reservoirs [97]. The damage control capabilities and sweep efficiency of the CO_2_ foam formulation during oil displacement are determined via comparative experimental analysis. To facilitate comparison with the anti-swelling CO_2_ foam flooding system, three distinct control groups are established: conventional water flooding, pure CO_2_ flooding, and conventional water-based CO_2_ foam flooding. The functionality of the anti-swelling foam system is attributed to dual mechanisms: first, formation permeability is preserved by the anti-swelling agent, thereby ensuring the injectivity and transportability of the foam. Second, viscous fingering and gravity override associated with CO_2_ are effectively inhibited by the foam; this mitigation is facilitated by its high apparent viscosity and the Jamin effect [98], significantly improving the microscopic oil washing efficiency and macroscopic sweep volume of CO_2_.

In general, as early as 1990, CO_2_ foam was utilized by Jonas et al. [99] to plug high-permeability layers in the Rangely Weber Sand Unit (northwestern Colorado), thereby improving sweep efficiency and CO_2_ utilization. Although the cost of CO_2_ injection was doubled by the foam application, it was determined to be lower than that of alternative profile control techniques, such as polymer gels. The engineering feasibility and economic viability of the modified water-based anti-swelling CO_2_ foam flooding system are identified as its primary advantages. Established protocols for water-based foam are adhered to by this system, while the highest degree of technical maturity and relatively controllable chemical costs are simultaneously exhibited. Consequently, this method is currently regarded as the “low-damage” foam flooding technical trajectory most proximal to field industrial application [75,76,77]. Concurrently, technical deficiencies persist within current methodologies. Specifically, the optimization of injectivity and anti-swelling properties is necessitated, while chemical compatibility costs remain elevated. Furthermore, long-term stability profiles are currently deemed insufficient to meet the rigorous demands of extended EOR engineering projects. Consequently, it is recommended that future research focus on the development of novel anti-swelling agents capable of simultaneously satisfying critical criteria: low molecular weight, robust adsorption, high resistance to leaching, and optimal compatibility with foaming agents. Alternatively, the investigation of “integrated” amphoteric surfactants featuring specialized molecular structures is proposed; such agents are envisioned to concurrently fulfill anti-swelling requirements while guaranteeing superior foam performance.

## 4. CO_2_ Organic Emulsion Foam Flooding System

### 4.1. Principle of System Construction

The fundamental basis of this system is constituted by the utilization of oil-soluble surfactants within organic solvents, with supercritical CO_2_ (scCO_2_) functioning as an auxiliary phase. With the primary objective of bypassing the osmotic swelling described in Section 2.2.3, this system fundamentally avoids water-induced corrosion and sensitivity by substituting the aqueous phase. Despite the inherently low viscosity exhibited by supercritical CO_2_ (scCO_2_), foam emulsions are generated in situ by surfactants dissolved within an organic solvent carrier upon interaction with residual water or oil phases. Through this process, a reduction in Interfacial Tension (IFT) and an enhancement of foam half-life are simultaneously effected [100]. Specifically, the elasticity of the foam film is enhanced via the Pickering stabilization mechanism [101]. High-permeability channels are effectively occluded. Concurrently, hydrophilic interactions with swelling clays are minimized by the low polarity of the organic phase, whereby the clay swelling rate is controlled at <5% [102]. System stability is further reinforced by foam-stabilizing polymers. Specifically, the inhibition of foam rupture is achieved via the elevation of solution viscosity (typically reaching 10–50 cP). The thermodynamic conditions requisite for the hydration swelling mechanisms (as delineated in Section 2.2) are effectively eliminated via the substitution of the continuous aqueous phase with an organic solvent, thereby achieving control over the occurrence of water sensitivity damage [103]. In this system, the continuous phase of the foam is constituted by an emulsion. The external phase of this emulsion is non-aqueous (organic solvent), while CO_2_ functions as the dispersed internal phase. Simultaneously, trace amounts of formation fluids are encapsulated within the organic solvent, thereby forming minute emulsion droplets dispersed within the continuous phase. Consequently, contact with the rock pore walls is restricted exclusively to the non-damaging organic phase, whereby water sensitivity damage is physically eradicated. Through the rapid formation of organic emulsions and the subsequent generation of high-strength CO_2_ foam, preferential channel plugging, sweep volume expansion, and enhanced oil displacement efficiency are effectively achieved.

### 4.2. System Composition

#### 4.2.1. Selection of Organic Phase

Solvents utilized within this category typically encompass light hydrocarbons (C5–C8), condensate oil, diesel, kerosene, and specific alcohols or esters [103,104,105]. Operational costs are significantly reduced through the utilization of refinery by-products (e.g., diesel and condensate). Furthermore, the solvent is required to exhibit miscibility or near-miscibility with CO_2_ at reservoir pressure; this property facilitates viscosity reduction and promotes the dissolution of heavy crude components (such as wax and asphaltenes).

#### 4.2.2. Selection of Emulsifiers and Foaming Agents

Oil-solubility is a prerequisite for the selected agents; typical examples encompass fluorocarbon, silicone, or specific anionic and non-ionic surfactant formulations [106,107]. Alternatively, the utilization of solid surfactant particles exhibiting reduced particle size and superior dispersibility within the organic phase is proposed. Through this approach, highly efficient and stable emulsions, alongside foams of robust stability, are achieved. Within the context of water-sensitive shale oil reservoirs, mixed Pickering foams are formed via the integration of similar non-aqueous formulations with nanoparticles (such as SiO_2_ solid particles). Consequently, the capillary force balance is optimized, while plugging induced by asphaltene precipitation is significantly mitigated [108].

### 4.3. Applications and Verification

The generation mechanism of foam, its rheological behavior, and its influence on oil displacement efficiency were investigated by Sie et al. [104] via foam stability tests and microfluidic model experiments. Non-aqueous foam was applied to hydrocarbon miscible flooding, wherein its generation and transport mechanisms were elucidated through pore-scale visualization. Consequently, an environmentally friendly and efficient novel EOR method for water-sensitive tight oil reservoirs was proposed, whereby problems associated with water injection are effectively circumvented. Non-aqueous foam was prepared by Li et al. [105,109] utilizing two oil-soluble fluorocarbon surfactants (FS22 and FSG). A comparative analysis was conducted against a control group consisting of water-phase foam stabilized by alkyl polyglucosides (APG0810), underscoring the critical influence of surface dilatational rheology and liquid film thinning behavior on foam stability. Stability within oil-containing environments was maintained by the non-aqueous foam via high viscoelasticity and a retarded liquid film drainage mechanism. Notably, superior stability was exhibited by FS22, a result attributed to its lower surface tension and higher viscoelastic modulus. Four distinct methodologies—namely CO_2_ flooding, CO_2_ huff-n-puff, oil-based CO_2_ foam flooding, and oil-based CO_2_ foam huff-n-puff—were compared by Wang et al. [110] via one-dimensional flow simulation experiments. Low-permeability sandstone cores, designed to simulate the characteristics of the Q131 block in the Liaohe Oilfield, were utilized. Concurrently, the spatial distribution of residual oil saturation within the core matrix was analyzed via Nuclear Magnetic Resonance (NMR) technology. It was demonstrated that crude oil recovery is significantly improved, and carbon sequestration efficiency is enhanced, by the oil-based CO_2_ foam system. Consequently, the efficacy of this method in providing the dual advantages of Enhanced Oil Recovery (EOR) and Carbon Capture, Utilization, and Storage (CCUS) within low-permeability reservoirs was confirmed. A composite surfactant (designated as SF) was formulated by Li et al. [106] through the compounding of Span 20 and the fluorocarbon surfactant F-1. At a concentration of 1.0 wt%, optimal stability was demonstrated, characterized by an oil-based foam volume of 275 mL and a half-life of 302 s. Consequently, the formation damage typically associated with water-based foams in water-sensitive reservoirs was circumvented by the oil-based system, which simultaneously exhibited the dual advantages of minimized formation damage and corrosion control. Furthermore, laboratory experiments were conducted to compare the efficacy of pure CO_2_ displacement against CO_2_ oil-based foam displacement within both fractured and non-fractured low-permeability cores. Nuclear Magnetic Resonance (NMR) imaging revealed that following pure CO_2_ displacement, residual oil remained predominantly concentrated within small pores and at the distal end of the core. In contrast, a lower and more uniformly distributed residual oil saturation was observed following CO_2_ oil-based foam displacement; this indicates that CO_2_ mobility was effectively controlled and sweep volume expanded by the foam.

### 4.4. Performance Evaluation Methodology

**Figure 5:**
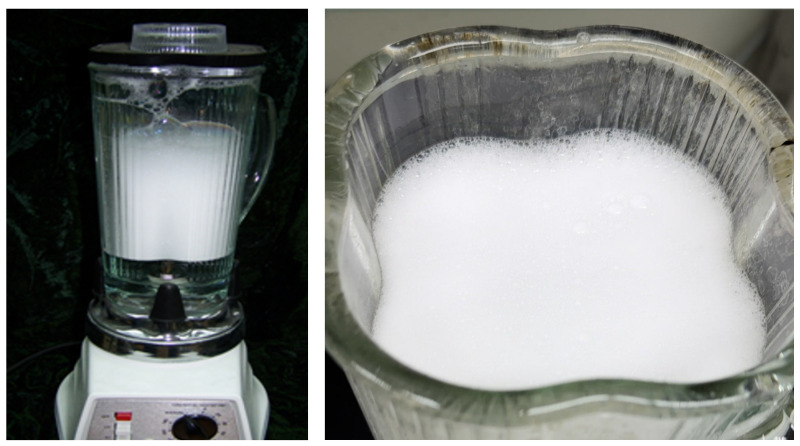
Preparation of CO_2_ organic emulsion foam via the stirring method under continuous CO_2_ injection.

The evaluation of the CO_2_ organic emulsion foam flooding system encompasses the assessment of foam performance characteristics [94], the evaluation scope extends to the determination of formation damage severity and the execution of oil displacement experiments. To verify the system’s formation capability, the CO_2_ organic emulsion foam was prepared via the stirring method under continuous CO_2_ injection. Figure 5 presents the visual observation of the resulting emulsion, where a stable, thick foam layer is formed, effectively isolating the water phase. When the organic emulsion system within the CO_2_ organic emulsion foam flooding framework is optimally designed, ultra-low interfacial tension levels (<10^−3^ mN/m) with formation crude oil may be achieved. In this capacity, the organic emulsion functions as a highly efficient surfactant system; consequently, capillary-trapped oil—inaccessible to conventional water or gas flooding—is effectively mobilized (“washed out”) via mechanisms of emulsification and solubilization. Accordingly, specific oil washing experiments are designed to quantitatively evaluate this mobilization efficacy.

In general, the fundamental challenges associated with the development of strongly water-sensitive reservoirs are addressed by the advantages inherent in the CO_2_ organic emulsion foam flooding system. Extremely high theoretical oil displacement efficiency is achieved through a multi-mechanism synergy, wherein the benefits of foam profile control, CO_2_ flooding, solvent flooding, and emulsion flooding are combined. However, technical difficulties persist, primarily regarding the high economic cost of organic solvents. Consequently, the screening of cost-effective organic solvents or the development of efficient solvent recovery processes is necessitated to mitigate economic expenditures. Furthermore, maintaining the long-term stability of the emulsion under conditions of high temperature, high pressure, high shear, and high salinity formation water contamination constitutes a significant chemical challenge. The viscosity of the emulsion is very high (generally > 100 cP, at >50 °C, The shear rate is 7.338 s^−1^). Despite the viscosity reduction facilitated by CO_2_ dissolution, the injectivity of high-viscosity emulsions within “tight” reservoirs is identified as a persistent and significant challenge. Consequently, it is recommended that future research focus on the utilization of bio-based organic solvents and switchable (CO_2_-responsive) surfactants. Furthermore, the integration of these materials with numerical simulations (e.g., foam flow models) is advocated to rigorously evaluate deep transport efficiency.

## 5. CO_2_-Soluble Foam Flooding System

### 5.1. Principle of System Construction

By eliminating the water phase, this system entirely circumvents the hydration mechanisms described in Section 2.2. Fundamentally, it treats CO_2_ not as a gas, but as a solvent in its supercritical or near-critical state (>7.38 MPa, >31.1 °C) [111,112]. Supercritical CO_2_ (scCO_2_) is characterized as a tunable non-polar solvent exhibiting a density comparable to that of a liquid, a viscosity analogous to that of a gas, and a high diffusion coefficient. Furthermore, its solvent strength is controlled via the modulation of pressure and temperature [113]. Through molecular design, CO_2_-philic surfactants capable of direct dissolution in Supercritical CO_2_ (scCO_2_) are synthesized, utilizing CO_2_ itself as the continuous phase and solvent. Consequently, water is precluded as the continuous phase of the foam, and its utilization is minimized. Trace quantities of water (originating from micro-injection or formation water) are sequestered by the CO_2_-philic surfactants, resulting in the formation of “W/scCO_2_” emulsions dispersed within the continuous CO_2_ phase. The free water phase is fundamentally eliminated by this system; thereby, water sensitivity damage is completely circumvented. Simultaneously, the expansion of the CO_2_ flood sweep volume and the enhancement of crude oil recovery are achieved [13].

### 5.2. System Composition

Conventional surfactants are characterized as “oil/water” amphiphilic; consequently, complete insolubility within non-polar CO_2_ is exhibited. Therefore, the establishment of a CO_2_-soluble foam system is entirely contingent upon the successful design of “CO_2_/water” or “CO_2_/oil” amphiphilic molecules possessing high solubility within the CO_2_ phase.

The design concept of “CO_2_-philicity” is predicated on the identification of a “tail” moiety exhibiting favorable thermodynamic interactions with CO_2_ (specifically, low cohesive energy density and favorable Lewis acid-base interactions) [114,115]. CO_2_ is a non-polar solvent with an extremely low dielectric constant (ε ≈ 1.5), and its “solvation” capability is far weaker than that of water (ε ≈ 80). Therefore, the critical micelle concentration (CMC) required for surfactants to form micelles in CO_2_ is far higher than their CMC in water [112]. Consequently, to achieve an effective concentration, the utilization of a substantial dosage of CO_2_-soluble surfactants is necessitated, thereby resulting in elevated operational costs.

**Figure 6:**
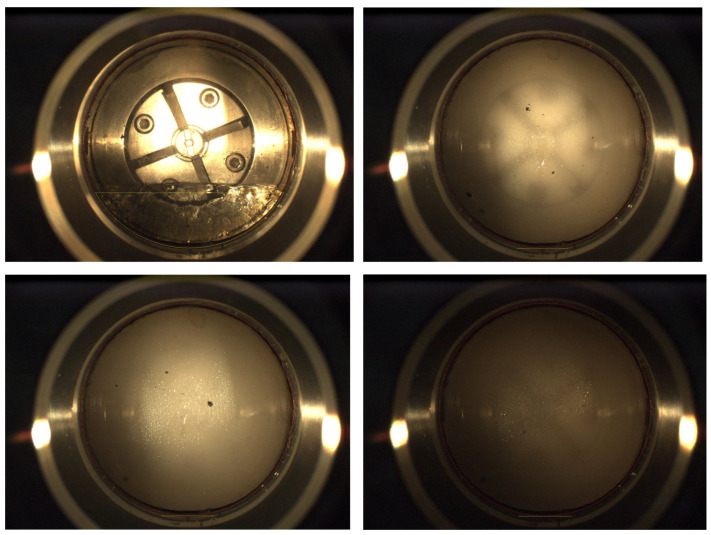
Phase behavior transition and foam generation of the CO_2_-soluble surfactant system observed in a high-pressure visual cell.

Surfactant solubility within CO_2_ is typically characterized by the “Cloud Point Pressure.” At a specified temperature, superior solubility within CO_2_ is indicated by a lower cloud point pressure [116]. Attributed to the extremely low polarizability and weak van der Waals forces characteristic of fluorine atoms, fluorocarbon chains within fluorocarbon surfactants are recognized as the most effective CO_2_-soluble moieties. Consequently, early research efforts were predominantly focused on perfluoropolyether surfactants, which exhibit the capacity to dissolve and generate robust foam even at notably low pressures [117]. However, the viability of these materials for Enhanced Oil Recovery (EOR) applications is effectively negated by their prohibitive economic costs and the persistent environmental contamination associated with per- and polyfluoroalkyl substances (PFAS) [118]. As alternatives to fluorinated compounds, siloxane surfactants are distinguished by their relatively favorable CO_2_ solubility and moderate economic costs. Interactions between surfactant molecules are effectively impeded by siloxane surfactants possessing short and branched “Stubby” hydrophobic tail moieties; consequently, solubility within CO_2_ is significantly enhanced. Furthermore, it is indicated by various studies that microemulsions or foams may be formed by specific siloxane surfactants in the presence of minimal water content [119]. Four siloxane polyether surfactants exhibiting varying polyethylene glycol chain lengths—specifically (PEG)_3_-TS, (PEG)_7_-TS, (PEG)_12_-TS, and (PEG)_16_-TS)—were synthesized by Shi et al. [120] via a two-step methodology. Solubility pressures were subsequently measured utilizing a high-pressure visual cell within a temperature range of 318–343 K and a pressure range of 8–20 MPa, allowing for the calculation of their solubility. It was confirmed by this study that siloxane polyether surfactants possess excellent solubility properties within supercritical CO_2_ (scCO_2_). The phase behavior is a critical indicator of solubility. As illustrated in Figure 6, the phase transition and foam generation of the CO_2_-soluble surfactant system were observed in a high-pressure visual cell. The disappearance of the clear interface confirms the formation of a homogeneous phase or micro-emulsion suitable for injection. However, analogous to fluorocarbon surfactants, prohibitive economic costs and toxicity concerns are also associated with siloxane surfactants. Consequently, contemporary research endeavors are principally directed toward the application of fluorine-free and silicon-free surfactant systems. Within this context, hydrocarbon non-ionic surfactants have emerged as a prominent area of investigation, with particular emphasis placed on ethoxylated amines and specific non-ionic formulations [121]. The phase behavior of a multi-component system—comprising supercritical CO_2_ (scCO_2_), non-ionic ethoxylated surfactants (specifically IGEPAL CO-630 and CO-720), and methanol—was investigated by Moacir et al. [122]. It was demonstrated that the inclusion of methanol as a co-solvent effectively enhances solubility within the CO_2_ phase. Non-ionic surfactants (exemplified by TDA-11) were dissolved in CO_2_ by Burrows et al. [123] to facilitate in situ foam generation. Consequently, the sweep efficiency of CO_2_ within the formation was significantly improved, while sequestration efficiency was concurrently enhanced. A branched alcohol polyoxyethylene ether (X1320) and a non-ionic alkyl polyglucoside surfactant were utilized by Liu et al. [116] Upon increasing the surfactant concentration from 0%to 1.2%, a significant decrease in the CO_2_-oil/water interfacial tension was observed. Concurrently, the minimum miscibility pressure (MMP) was reduced from 31.4 MPa to 27.6 MPa, thereby improving the miscibility efficiency between CO_2_ and crude oil. Furthermore, research has identified the utility of “Switchable Surfactants” (specifically amine-based compounds), which employ CO_2_ as a functional trigger to transition between non-ionic (soluble in CO_2_) and cationic (soluble in water) states. Upon dissolution in CO_2_ and subsequent injection into the formation, these agents protonate upon contact with the aqueous phase, transitioning to a cationic form. This transformation not only facilitates foam generation but also enables the surfactant to function as an anti-swelling stabilizer. The utilization of a novel alkyl amine surfactant was reported by Cui et al. [124], wherein superior performance was exhibited. Specifically, stable dissolution within both aqueous and CO_2_ phases was maintained under rigorous conditions, including high temperature (110 °C), high salinity (220,000 ppm NaCl), and acidic environments. Furthermore, low adsorption characteristics were demonstrated, alongside the capacity to rapidly generate robust CO_2_ foam and emulsions. A novel dynamic adsorption testing method was developed by Wu et al. [125] to evaluate a CO_2_-soluble surfactant (Duomeen CTM) under reservoir conditions. It was demonstrated that the apparent viscosity and sequestration efficiency of CO_2_ are significantly increased by this surfactant. Molecular dynamics (MD) simulations were integrated with experimental verification by Zhang et al. [126] to investigate the enhancement of anionic surfactant AOT solubility in supercritical CO_2_ via ethanol as a co-solvent, alongside its subsequent impact on foam stability. MD simulations were employed to elucidate the mechanism by which ethanol improves AOT solubility; specifically, by weakening inter-surfactant molecular interactions and enhancing the solvation affinity between AOT and the CO_2_/ethanol solvent mixture. Furthermore, the influence of ethanol on the CO_2_/water interfacial characteristics—including interfacial thickness, tension, and intermolecular forces—was quantitatively analyzed.

Molecular dynamics simulations conducted by Kobayashi et al. [127] indicated that an increased density of methyl (CH_3_) groups within branched alkanes results in a higher coordination number with CO_2_. This leads to enhanced solvation affinity between the alkanes and CO_2_, thereby improving solubility. Molecular-level guidance for the design of efficient CO_2_ chemical agents is provided by the optimization of hydrocarbon solubility in CO_2_ via structural regulation, such as increased branching.

A distinct trade-off is revealed upon comparing the three classes of CO_2_-philic surfactants: while the highest solubility and lowest miscibility pressures are offered by fluorocarbon-based agents [117], their application is constrained by prohibitive environmental regulations (PFAS restrictions). Siloxane-based surfactants represent a performance middle ground; however, their economic viability remains limited by high production costs. Consequently, a shift is observed in industry trends toward hydrocarbon-based non-ionic surfactants [121,123], wherein a reduction in CO_2_ solubility is accepted in exchange for significant cost reductions and superior environmental compliance. Future success is contingent upon the molecular optimization of ‘stubby’ tail architectures within these hydrocarbon systems to rival the performance of fluorinated counterparts.

### 5.3. Generation and Stability Mechanism

CO_2_-philic surfactants are characterized by their CO_2_/water amphiphilicity. Upon the encounter of surfactant molecules—dissolved within the continuous supercritical CO_2_ phase—with trace quantities of water (derived from formation water or minor injected volumes), spontaneous aggregation at the CO_2_/water interface is observed. In this configuration, CO_2_-philic tails are oriented into the CO_2_ phase, while hydrophilic head groups extend into the aqueous phase. As the continuous sc CO_2_ phase migrates and contacts bound or trace-injected water within the formation, the surfactant is partitioned to the CO_2_/water interface. Consequently, a high-quality foam is generated, wherein a minimal volume of water serves as the liquid lamellae and CO_2_ constitutes the internal gas core. Alternatively, “water-in- CO_2_” microemulsions (W/sc CO_2_) may be formed; these primarily function to facilitate viscosity enhancement (CO_2_ thickening) rather than traditional foam-based mobility control [124].

### 5.4. Damage Control and Potential

#### 5.4.1. Damage Control

Non-aqueous scCO_2_ is utilized as the continuous phase. Water is sequestered within micro-emulsion droplets, thereby facilitating its spatial isolation from clay minerals. Consequently, the thermodynamic and kinetic conditions necessary for clay swelling and fines migration are effectively precluded.

#### 5.4.2. Potential

The CO_2_-soluble foam system enhances oil displacement through two primary mechanisms: microscopic miscibility extraction and macroscopic mobility control. First, supercritical CO_2_ (scCO_2_) achieves oil displacement via extraction and miscibility mechanisms, effectively mobilizing crude oil in nanopores [113]. Second, regarding mobility control, the high flow resistance inherent in the CO_2_-soluble foam system necessitates flow redirection. This forces CO_2_ to uniformly sweep medium-to-low permeability zones. Consequently, the gravity override and viscous fingering phenomena—common in conventional CO_2_ flooding—are effectively mitigated. This system is specifically optimized for tight reservoirs characterized by significant heterogeneity and the presence of micro-fractures. Preferential flow channels are effectively obstructed by the foam, thereby compelling CO_2_ to penetrate tight zones and exert its microscopic oil displacement advantages.

A tripartite integration of zero water-sensitivity damage, maximized CO_2_ utilization, and superior conformance control is achieved by the CO_2_-soluble foam system for strongly water-sensitive tight oil reservoirs. Primary technical challenges are identified as the high synthesis costs and complexities of surfactants. Furthermore, the mitigation of high critical micelle concentrations (CMC) necessitates the incorporation of suitable co-agents, while long-term stability and adsorption kinetics require further exploration. Additionally, the optimization and upgrading of field injection equipment and processes are required to facilitate the uniform surface mixing of solid/liquid surfactants with supercritical CO_2_ under high-pressure conditions. Future research efforts should be directed toward the development of cost-effective hydrocarbon surfactants—specifically, high-efficiency, fluorine-free, and silicon-free variants derived from inexpensive raw materials such as polyethers or branched alcohols. Alternatively, the design of intelligent or switchable surfactants is advocated; These agents remain water-soluble to facilitate surface injection. Upon contact with supercritical CO_2_ within the formation, they undergo in situ chemical transitions to become CO_2_-philic [128,129]. The implementation of this “switchable” technology possesses the potential to address both prohibitive economic expenditures and operational injection challenges.

## 6. The Application and Selection Guidelines for CO_2_ Foam Flooding

### 6.1. Field Application

In recent years, CO_2_ foam flooding technology has progressed from laboratory research to field application. Following systematic experimental screening and performance evaluation, a formulation for a high-temperature-tolerant, salt-resistant, low-adsorption, and highly stable nanoparticle-stabilized CO_2_ foam flooding system was developed by Liu et al. [130] for the Shu16 Block of the Yushulin Oilfield. It was demonstrated that this system not only reduces oil–water interfacial tension (IFT) and generates foam to enhance displacement efficiency in macro-pores, but also utilizes CO_2_ to accelerate the demulsification of heavy crude oil. Consequently, a critical experimental foundation and technical support are provided by these findings for the field application of this foam system in low-permeability reservoirs. A CO_2_ foam flooding system was developed by Wang et al. [131] specifically for high-temperature (97.3 °C), low-permeability reservoirs in the H79 Block of the Jilin Oilfield, China. This system was designed to address challenges such as ineffective water flooding, severe gas channeling during CO_2_ injection, and poor sweep efficiency, thereby significantly enhancing the crude oil recovery factor. CO_2_ foam was utilized by Patilet al. [132] in the sandstone reservoir of the Salt Creek Field, located in Natrona County, Wyoming. In comparison to the baseline Water-Alternating-Gas (WAG) prediction, a cumulative increase in crude oil production of approximately 25,000 barrels was achieved by the foam pilot project. It was indicated by tracer tests that gas mobility was reduced by the foam, thereby improving CO_2_ sweep efficiency. To address the challenge of low CO_2_ sweep efficiency during tertiary CO_2_ miscible flooding in the heterogeneous carbonate reservoirs of the East Seminole Field (Permian Basin, TX, USA), the Surfactant-Alternating-Gas (SAG) technique was employed by Paul Alcorn et al. [133]. It was demonstrated that CO_2_ mobility was successfully reduced by the foam, thereby improving sweep efficiency and enhancing crude oil recovery even with a reduced injection volume. Ultimately, the effectiveness of foam application in both CO_2_ Enhanced Oil Recovery (EOR) and carbon sequestration processes was confirmed by the field data. It was emphasized by Yuan et al. [134] in their review that foam-assisted WAG possesses a significant capacity to reduce the gas-oil ratio (GOR) and suppress gas breakthrough, thereby substantially enhancing CO_2_ displacement efficiency. Subsequently, a pilot test of foam-assisted WAG was conducted in the Black 125 Block of the Jilin Oilfield, from which favorable initial results were obtained, demonstrating the potential of this technology to improve CO_2_ displacement performance. It was reported by Lyu et al. [15] in their review that a pilot test of foam-assisted enhanced displacement was conducted in the Hei-125 Block of the Jilin Oilfield, China. Positive results were achieved, characterized by increased fluid and oil production rates, alongside a concurrent reduction in the gas-oil ratio (GOR).

The substantial application potential of CO_2_ foam has been demonstrated by numerous field tests. However, to date, there are no reports in the literature regarding the specific application of CO_2_ foam flooding in highly water-sensitive tight oil reservoirs. It is anticipated that CO_2_ foam flooding technology can significantly mitigate challenges such as premature breakthrough and poor sweep efficiency associated with CO_2_ injection, while simultaneously minimizing formation damage caused by water sensitivity. By leveraging these advantages, the development of unconventional resources, such as tight oil reservoirs, can be substantially enhanced.

### 6.2. Selection Guidelines

The water-based anti-expansion CO_2_ foam flooding system, CO_2_ organic emulsion foam flooding system and CO_2_ soluble foam flooding system can all maintain good foaming performance under high-temperature and high-salt reservoir conditions (temperature T < 90 °C, total dissolved solids TDS > 30,000 mg). All three systems need to exceed the critical pressure of CO_2_ (>7.38 MPa) to ensure they are supercritical CO_2_ (scCO_2_). The balance between anti-swelling performance and injectivity should be found through experimentation (generally Viscosity < 10 cP, at >50 °C, The shear rate is 7.338 s^−1^).

Other parameters are compared accordingly in this chapter. The comprehensive performance comparison of the three CO_2_ foam flooding systems is shown in Table 2.

## 7. Conclusions and Outlook

### 7.1. Conclusions

Water sensitivity damage in tight oil reservoirs originates from the lattice expansion and fines migration of clay minerals, and its damage to permeability is fatal and difficult to reverse. Three low-damage CO_2_ foam flooding systems, namely the modified water-based anti-swelling CO_2_ foam system, CO_2_ organic emulsion foam system, and CO_2_-soluble foam system, represent defense strategies at three levels: the modified water-based anti-swelling CO_2_ foam system “passivates” clay through chemical agents; the CO_2_ organic emulsion foam system “physically isolates” the water phase through organic media; and the CO_2_-soluble foam system achieves “minimal water/waterless” operations through phase state control.

The modified water-based anti-swelling CO_2_ foam flooding system represents the most pragmatic contemporary choice, as it possesses the highest level of technical maturity and superior economic feasibility. Through the introduction of novel small-molecule quaternary ammonium salts or low-molecular-weight polymers, the clay swelling rate is significantly attenuated without necessitating modifications to existing water injection protocols. However, long-term resistance to washout and the charge compatibility between anti-swelling and foaming agents are identified as the primary constraints hindering large-scale field application

The CO_2_ organic emulsion foam flooding system is identified as a specific remediation strategy for reservoirs characterized by extreme water sensitivity or water-lock phenomena. The inducement of water sensitivity is fundamentally eliminated through the utilization of oil or organic solvents as the continuous phase. Furthermore, auxiliary benefits, including the removal of water locks and the optimization of wettability, are provided by this system. While the formation of an emulsion addresses the issue of reservoir damage, large-scale implementation is currently constrained by prohibitive solvent costs and complex surface preparation protocols. Consequently, the application of this system is considered most appropriate for niche, high-value-added oilfield operations.

The CO_2_-soluble foam flooding system is identified as the primary trajectory for future research; the direct dissolution of surfactants into supercritical CO_2_ for injection is considered a paradigm shift in fluid transport technology. Through this approach, zero water-sensitivity damage is ensured, while the geological sequestration potential of CO_2_ (CCUS) is maximized. The primary contemporary bottleneck is identified as the development of cost-effective, environmentally sustainable, and highly soluble non-fluorinated CO_2_-philic surfactants (e.g., hydrocarbon- or siloxane-based systems).

In summary, the comparative analysis highlights distinct application scenarios for each system based on the “Compatibility Paradox.” Modified water-based systems offer the highest economic viability for reservoirs with moderate water sensitivity, provided cationic stabilizers are compatible with foaming agents. Conversely, organic emulsion systems provide the ultimate “physical isolation” defense for high-value, ultra-sensitive reservoirs, albeit at a higher chemical cost. Finally, CO_2_-soluble systems represent the transformative solution for ultra-tight formations, unlocking the potential for true “waterless” stimulation and maximizing CO_2_ storage capacity despite current surfactant cost challenges.

### 7.2. Outlook

To facilitate the scalar transition of low-damage CO_2_ foam technology toward industrial application within tight oil reservoirs, future research priorities are identified as follows:

The design and development of novel small-molecule quaternary ammonium salts or low-molecular-weight polymers are required, with an emphasis on creating environmentally sustainable and cost-effective CO_2_-philic materials. Presently, CO_2_-soluble surfactants are predominantly reliant upon expensive fluorocarbon or siloxane chains. Future breakthroughs are anticipated in the development of hydrocarbon-based or oxygen/ester-containing CO_2_-philic surfactants. Molecular dynamics (MD) simulations should be utilized to facilitate the structural optimization of branches and the distribution of polar groups, thereby significantly reducing synthesis costs to <5000$/ton to ensure economic competitiveness with conventional chemical agents, and ensure that the cost for each additional barrel of oil is less than 55$.

The development of surfactants with switchable functionalities is required to facilitate the construction of intelligent responsive fluids. These molecules are designed to remain water-soluble under surface conditions, thereby facilitating fluid preparation. Upon reservoir entry, they are triggered by CO_2_ partial pressure to undergo an in situ transformation into a CO_2_-philic or cationic anti-swelling state. This approach is expected to address the compatibility challenges inherent in modified water-based anti-swelling CO_2_ foam flooding systems, as well as the operational constraints associated with injection equipment in CO_2_-soluble foam flooding systems.

Future evaluation frameworks must expand beyond enhanced oil recovery (EOR) metrics to incorporate CO_2_ sequestration efficiency. The long-term impacts of geochemical evolution—specifically the reactions occurring between clay minerals and CO_2_—must be rigorously investigated to determine their influence on reservoir integrity and foam stability within highly water-sensitive formations.

## Figures and Tables

**Table 1:** Cation Exchange Capacity of Common Clay Minerals [47].

Clay Minerals	Cation Exchange Capacity/(meq/100 g)
kaolinite	0–3
montmorillonite	10–20
illite	3–20
chlorite	5–20

**Table 2:** Comparison of Comprehensive Performance of Three Low-Damage CO_2_ Foam Flooding Systems.

Evaluation Dimension	Water-Based Anti-Swelling CO_2_ Foam	CO_2_ Organic Emulsion Foam	CO_2_-Soluble Foam
Water Sensitivity Resistance	Moderate (Relies on chemical inhibition, risks remain)	Extremely High (Physical isolation)	High
Mobility Control Ability	Strong (Foam quality easy to control)	Medium-Strong (Limited by oil phase viscosity)	Strong (In situ foaming, good deep plugging)
Chemical Cost	Low(<$45/bbl)	High (<$55/bbl)	Medium-High (<$55/bbl)
Operation Process	Simple (Conventional water injection equipment)	Moderate (Requires explosion-proof, oil circulation systems)	Simple (Single string injection)
Environmental Impact	Medium (Flowback fluid treatment required)	High (Risk of organic leakage, Use environmentally friendly organic solvents.)	Low
Applicable Reservoirs	Permeability > 1 mD, clay content V_sh_ < 15%	Strong water sensitivity, I/S mixed-layer content > 50%, Permeability < 10 mD	Ultra-low permeability/shale, reservoirs with developed micro-fractures, Permeability < 0.3 mD

## Data Availability

No new data were created or analyzed in this study. Data sharing is not applicable to this article.
